# Hippocampal sclerosis, hippocampal neuron loss patterns and TDP‐43 in the aged population

**DOI:** 10.1111/bpa.12556

**Published:** 2017-09-25

**Authors:** Suvi R. K. Hokkanen, Sally Hunter, Tuomo M. Polvikoski, Hannah A. D. Keage, Thais Minett, Fiona E. Matthews, Carol Brayne, MRC CFAS and CC75C Study Group

**Affiliations:** ^1^ Institute of Public Health University of Cambridge Cambridge UK; ^2^ Institute of Neuroscience Newcastle University Newcastle upon Tyne UK; ^3^ Cognitive Ageing and Impairment Neurosciences Laboratory, Social Work and Social Policy University of South Australia Adelaide South Australia; ^4^ Department of Radiology University of Cambridge Cambridge UK; ^5^ Institute for Health and Society Newcastle University Newcastle upon Tyne UK

**Keywords:** hippocampus, hippocampal sclerosis, neuron loss, population study, TDP‐43

## Abstract

Hippocampal neuron loss is a common neuropathological feature in old age with various underlying etiologies. Hippocampal sclerosis of aging (HS‐Aging) is neuropathologically characterized by severe CA1 neuronal loss and frequent presence of transactive response DNA‐binding protein of 43 kDa (TDP‐43) aggregations. Its etiology is unclear and currently no standardized approaches to measure HS‐Aging exist. We developed a semi‐quantitative protocol, which captures various hippocampal neuron loss patterns, and compared their occurrence in the context of HS‐Aging, TDP‐43, vascular and tau pathology in 672 brains (TDP‐43 staining n = 642/672, 96%) donated for the population‐based Cambridge City over‐75s Cohort and the Cognitive Function and Ageing Study. HS‐Aging was first evaluated independently from the protocol using the most common criteria defined in literature, and then described in detail through examination of neuron loss patterns and associated pathologies. 34 (5%) cases were identified, with a maximum of five pyramidal neurons in each of over half CA1 fields‐of‐view (x200 magnification), no vascular damage, no neuron loss in CA2‐CA4, but consistent TDP‐43 neuronal solid inclusions and neurites. We also report focal CA1 neuron loss with vascular pathology to affect predominantly CA1 bordering CA2 (Fisher's exact, *P* = 0.009), whereas neuron loss in the subicular end of CA1 was associated with TDP‐43 inclusions (Fisher's exact, *P* < 0.001) and high Braak stage (Fisher's exact, *P* = 0.001). Hippocampal neuron loss in CA4‐CA2 was not associated with TDP‐43. We conclude that hippocampal neuron loss patterns are associated with different etiologies within CA1, and propose that these patterns can be used to form objective criteria for HS‐Aging diagnosis. Finally, based on our results we hypothesize that neuron loss leading to HS‐Aging starts from the subicular end of CA1 when it is associated with TDP‐43 pathology, and that this neurodegenerative process is likely to be significantly more common than “end‐stage” HS‐Aging only.

## Introduction

Originally, the term “hippocampal sclerosis” was used to describe epilepsy patients' hippocampal changes 50, which can include neuron loss and gliosis in the CA areas 1, 3 and 4, granule cell dispersion, and hypertrophic neurons especially in CA4, with relative preservation of CA2 and subiculum and lack aggregated proteins associated with neurodegeneration 6, 9, 10, 47. In contrast, hippocampal sclerosis of aging (HS‐Aging) is considered as a distinct, dementia‐related pathology 18, 31, 35, 56, 58, neuropathologically characterized by severe neuron loss and gliosis in the CA1 area. The subiculum may be affected, but CA4, CA3 and are CA2 spared 16, 41, 43. Due to its frequent association with transactive response DNA‐binding protein of 43 kDa (TDP‐43)‐positive cytoplasmic aggregations presence of TDP‐43‐pathology has recently been used as an additional criterion for HS‐Aging 5, 34.

HS‐Aging may also present with neurofibrillary tangles, amyloid plaques 25, 31, argyrophilic grains 8 and Lewy bodies 7. Diagnostic criteria of HS‐Aging have largely required exclusion of cases with severe hippocampal tau pathology 8, 14, 28, 42 to distinguish HS‐Aging from severe Alzheimer's disease cases. A vascular etiology for HS‐Aging has also been posited on account of the high metabolic rate and relatively deficient vascular supply 14 that may predispose CA1 neurons to hypoxia 39, but results on this hypothesis are conflicting 1, 16, 31, 35, 37, 38.

Currently no objective definition of HS‐Aging exists and, thus, a range of potentially distinct pathologies has been included within this term. In association with differing study settings, this has led to wide variation of HS‐Aging prevalence statistics, from 0.4% 1 to 26% 18 in those with dementia. This complicates the interpretation, comparison and generalizability of findings. Previous studies have focused on “end‐stage” HS‐Aging, while the changes related to HS‐Aging initiation and progression remain unknown. This study is the first to 1 characterize end‐stage HS‐Aging, its epidemiology and pathological associations in a large population‐based cohort and 2 describe detailed patterns of hippocampal neuronal loss in association with vascular and neurodegenerative pathologies in the population.

## Materials and Methods

### Study design

The Cambridge City over‐75s Cohort (CC75C) and the Medical Research Council (MRC) Cognitive Function and Ageing Study (CFAS) are two of the few existing longitudinal population‐based clinicopathological studies. The cohort profiles have been previously described 13, 19. In brief, CC75C started in 1985 and recruited 2610 individuals (response rate of 95%) aged 75 years and above through seven general medical practices in Cambridge, UK. Recruitment for the brain donation program started shortly after the second survey, resulting in 241 donations at the time of this study. The brain donor cohort is representative of the general population in regard to basic sociodemographic indicators 54. CFAS recruited 18 226 people aged 65 years and above through primary care general population lists in six sites in England and Wales (82% response rate). People who were selected into a 20% stratified sample from each center for detailed assessment, and all who underwent this assessment were eligible for the approach for brain donation. By the time of the current study, 562 CFAS brain donations had been collected. Brain donors are comparable to the population sample for most of the basic sociodemographic indicators, except for their older age at death 32.

### Neuropathological protocols

In both CC75C and CFAS, after death, brains were retained as soon as possible. Cerebrum was bisected in sagittal plane. One hemisphere was dissected coronally into 1 cm slices and frozen at −80°C. The other hemisphere was formalin‐fixed for at least six weeks and dissected coronally into 1 cm slices. Hippocampal (at the level of the lateral geniculate body), entorhinal cortex (at the level of the mammillary body), frontal, temporal, parietal and occipital lobe, basal ganglia, thalamus, pons, medulla, cerebellum and two midbrain tissue blocks were paraffin‐embedded. In CC75C, sections were cut at 9–10 micrometer (µm), in CFAS at 7–9 µm, and stained with hematoxylin and eosin at the respective study centers. Standardized neuropathological assessments were carried out blinded to any clinical data at the respective study centers by clinical neuropathologists.

Severe atherosclerosis, arteriolosclerosis, cerebral macroscopic infarcts and cortical microinfarcts were assessed as present or absent with hematoxylin‐eosin sides. Anti‐tau monoclonal antibody (CC75C: mAb 11.57, a gift from Prof. C. M. Wischik, University of Aberdeen 11; CFAS: AT8, recognizing Ser202/Thr205) was used to immunostain neurofibrillary tangles, neuritic plaques and dystrophic neurites. The sections were counterstained with Ehrlich's hematoxylin with diaminobenzidine as the chromagen. Ratings for tau‐reactive tangles and neuritic plaques per section were graded as none, mild, moderate or severe, according to the CERAD protocol 33. These sections were also used to determine the Braak staging 12. However, due to historical reasons, not all CFAS cases had a Braak stage assigned (n = 221). Neurofibrillary tangle pathology in those cases was categorized by using three scores: 1 = none and mild tau‐reactive tangle pathology in the hippocampus and entorhinal cortex and no tau pathology in the neocortex; 2 = moderate or severe hippocampal and entorhinal tangle pathology and up to mild neocortical tau scores; 3= hippocampus as in score 2 and moderate or severe tau pathology was found in the frontal, temporal, parietal or occipital cortex. These scores highly correlated with the recorded Braak stage (1 = Braak 0‐II, 2 = Braak III‐IV, 3 = Braak V‐VI) in CFAS (Spearman's *ρ* = 0.798, *P* < 0.001). In analysis, the tangle score was used as a Braak stage estimate if the Braak stage was not known.

The CERAD plaque score refers to the maximum severity of tau reactive neuritic plaques in the frontal, temporal, entorhinal and parietal cortex.

### Evaluation of the hippocampus and neuron loss scoring

Work on hippocampal neuron loss was piloted and established within the CC75C. Two sets of hematoxylin‐eosin‐stained hippocampal sections were available for evaluation from 235 of the total of 241 brains; one from the CERAD diagnostic set (ten µm), and the other from the CC75C TDP‐43 study (9 µm). Exclusions were due to missing slides and blocks being used up.

**Figure 1:**
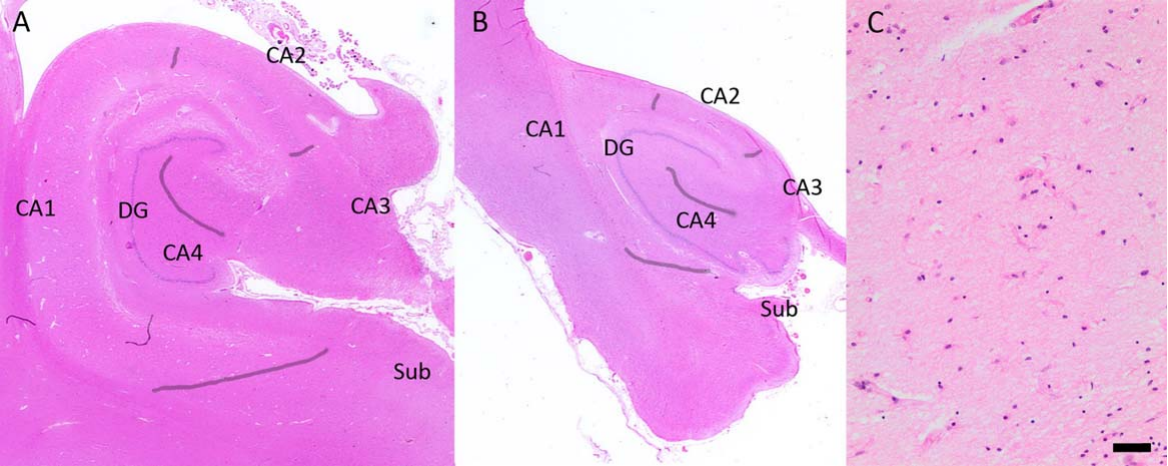
*HS‐Aging neuropathological characteristics on hematoxylin‐eosin‐staining*. **A.** Hippocampal formation without significant pathological findings. Hippocampal subfields are marked. **B.** A hippocampus with HS‐Aging shows severe atrophy and complete neuron loss, and gliosis in the whole CA1 area and subiculum (arrowheads). Other hippocampal areas appear intact. **C.** Microglia and pale eosinophilic astrocytes populate the CA1 region in HS‐Aging. Magnifications: (A, B): 2.5x; scale bar (C): 50 μm. DG = Dentate Gyrus, Sub = Subiculum.

Initial screening was performed under low (40x) and medium (100x) magnification. For each hippocampal region presence or absence of acute (tissue rarefication or astrocytosis and presence of eosinophilic anoxic neurons and white matter vacuolization) or older (sharply demarcated glial scars or cystic lesions) ischemic lesions was noted. For the diagnosis of HS‐Aging, SH and SRKH assessed independently, blinded to any clinical or other pathological data all available hippocampal hematoxylin‐eosin‐stained sections in CC75C, following the most common criteria in literature 16, 18, 36, 43: severe neuron loss and gliosis in CA1, where neuronal loss was not explained by an infarct, and no obvious neuron loss in other hippocampal areas was present (Figure 1). HS‐Aging was scored as either absent or present and both raters reached consensus on all cases. They were further reviewed by TMP for confirmation.

In the next step, neuron loss severity and extent in various parts of the hippocampus was evaluated in all cases, if the area was not beyond recognition e.g. due to an abscess or tumor. Apart from the subiculum, the term “neuron” in this study refers to Ammon's horn pyramidal neurons. The categories and workflow were optimized in several scoring rounds and discussions between SRHK, SH and TMP. Each pyramidal neuron was identified by its shape, size, position and visible nucleus. Of the hippocampal regions, CA4 was distinguished from CA3 by the location of CA4 neurons in the concave of the dentate gyrus and organization of neurons in CA3. The border between the narrowest region of the hippocampus CA2 and CA1 was determined by the widening of the pyramidal cell layer, and the morphology of cells changing from large ovoid compact CA2 neurons to triangular more scattered CA1 neurons. The transition from CA1 to the subiculum was identified through a narrow cell‐free zone between the regions, looser packing of the pyramidal cell layer in the subiculum than in CA1 and presence of smaller subicular neurons. The borders between hippocampal subfields are marked in Figure 1A,B. However, neuron populations frequently blend in transition zones between the CA areas. We followed the descriptions and illustrations by Amaral and Insausti 4 while defining hippocampal sectors.

Magnification of 200x was applied to assess neuron frequencies in all fields‐of‐view (ca 9.538 mm^2^) in the hippocampal regions (CA4, CA3, CA2, CA1, subiculum). In each region, neuron frequency was quantified in each field‐of‐view and assigned a semi‐quantitative severity score: 0–1 neuron; 2–5 neurons; 6–10 neurons; 11–15 neurons. Any neuron frequency, sufficient to reach the neuron loss severity benchmarks in a field‐of‐view, was scored independent from potential underlying causes.

**Figure 2:**
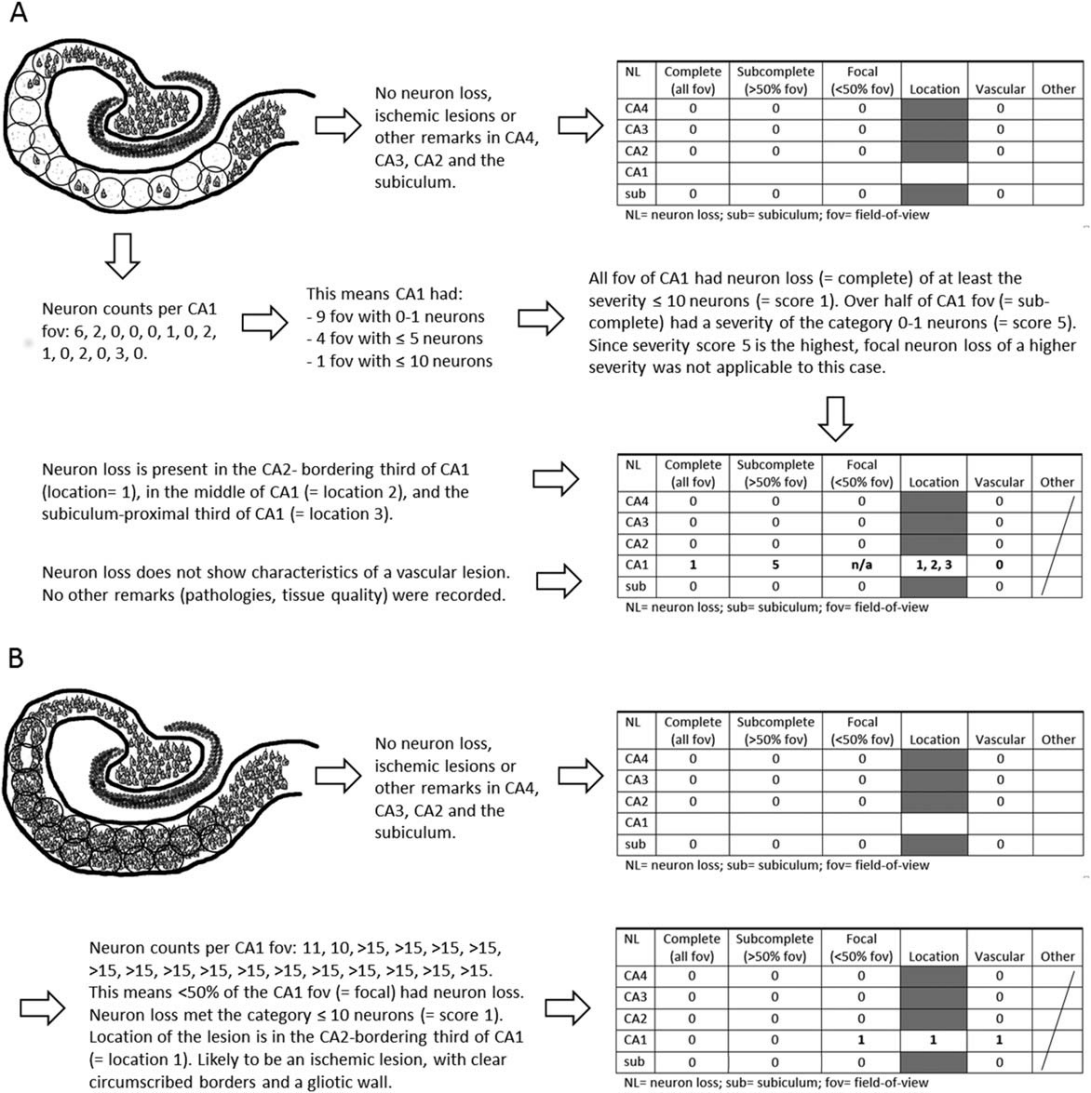
Schematic illustration of the hippocampal neuron loss scoring protocol workflow. Example (A) explains the scoring of a case with extensive severe neuron loss in CA1, and example (B) shows a case with a focal lesion in CA1 bordering CA2.

If neuron loss was detected, the extent was recorded for each severity in the following categories: complete = neuron loss affects every field‐of‐view at 200x magnification of the respective hippocampal area; sub‐complete = neuron loss affects over half of the fields‐of‐view in the respective area; focal = neuron loss in less than half of the fields‐of‐view in the respective area. If neuron loss in CA1 was not complete, its location was noted as beginning, middle or end. Borders of these subregions were based on the total number of fields‐of‐view used for the evaluation of the case's CA1. Several categories of neuron loss severity with differing extent could be marked for a case—not only for separate CA regions, but also for a subregion. Figure 2 illustrates schematically for two examples how neuron loss was scored and recorded.

Reliability and reproducibility of the protocol were tested through inter‐rater evaluation. All cases on both CC75C HE‐sets were first scored independently by two raters (SH, SRKH).

Next, the neuron loss assessment protocol was applied to the available 452 CFAS cohort cases. Excluded cases lacked routine neuropathological evaluation (= donations were too recent) or had missing hematoxylin‐eosin‐stained hippocampal slides with used‐up corresponding tissue blocks. Another 15 cases were omitted from final analysis, because tissue destruction, oxidation, or other technical shortcomings hindered the assessment, or because the case code was not identifiable. CFAS cases were assessed by SRKH, and results were inter‐rater evaluated on 10% (n = 45) by SH with near‐perfect agreement for all hippocampal neuron loss variables. All cases with neuron loss were reviewed and confirmed (TMP).

Following the neuron loss assessment, the previously diagnosed HS‐Aging cases from CC75C were characterized based on their neuron loss pattern. Finally, this characteristic neuron loss pattern was applied to the CFAS cohort to identify its HS‐Aging cases.

### Immunohistochemistry and assessment of TDP‐43

Hippocampal sections from 642 of 672 CC75C and CFAS cases that were evaluated for neuron loss, were included in the TDP‐43 assessment. Exclusions were due to the hippocampal tissue being used up. In 15 cases the entorhinal cortex could not be evaluated, mostly because the cortical structures were damaged/fragmented during tissue processing.

Hippocampal samples were immunostained with anti‐pTDP‐43 antibodies (Cosmo Bio Co LTD. pS409/410‐2, dilution 1:1000, antigen retrieval: EBSciences H2800 microwave processor, 15 mins at 98°C in a 0.01 M citrate buffer with pH 6.0). Anti‐pTDP‐43 immunoreactive neuronal solid cytoplasmic inclusions and short neurites were evaluated in the hippocampal area including dentate, CA4, CA3, CA2, CA1 and subiculum, and the entorhinal cortex by layer. The frequency of inclusions and neurites was scored for each area as none, single pathology, mild = inclusions/neurites seen in <50% fields‐of‐view, moderate = inclusions/neurites seen in >50% fields‐of‐view, frequent = inclusions/neurites seen in all or nearly all fields‐of‐view at 200x magnification. Scoring in CC75C was performed by SH and inter‐rater evaluated by SRKH, half of the scoring in CFAS was done by SH and half by SRKH, and inter‐rater evaluated reciprocally.

### Statistical analysis

Inter‐rater agreement was assessed calculating Gwet's Agreement Coefficient 2 (AC2), which is a paradox‐resistant alternative to Cohen's kappa 20, 21 when the overall present agreement is high. Inter‐rater analyses were performed blinded on 10% (CC75C: n = 23; CFAS: n = 45) of donated brains randomly selected but weighted according to severity scores. Analyses were performed using Agreestat 2015.1 (Advanced Analytics, Gaithersburg, MD, USA). The extent of agreement was evaluated using the benchmarks of Landis and Koch 30: >0.6 indicating substantial agreement and >0.8 near‐perfect agreement. Inter‐rater evaluation in both cohorts and for all hippocampal neuron loss and TDP‐43 variables reached near‐perfect agreement (AC2 (95% Confidence Interval [CI]) range: neuron loss: 0.83 (0.72–0.94) – 0.99 (0.98–1); TDP‐43 NSIC: 0.95 (0.87–1) – 0.99 (0.98–1), TDP‐43 neurites: 0.91 (0.83–0.98) – 1)).

For several association analyses, the severity of hippocampal TDP‐43 inclusions was dichotomized as present/absent. Scores for inclusions and neurites were respectively also collapsed for the hippocampus and dichotomized as absent in all hippocampal areas (dentate, CA4, CA3/2, CA1, subiculum) vs. present in any of these areas. Analogous, entorhinal cortex inclusions and neurites were respectively collapsed and dichotomized as absent in all entorhinal layers vs. present in any of the layers.

Continuous data were compared using the Student's test (*t*(degrees of freedom), 95%CI [mean difference]). Association between categorical variables were tested using the Chi‐square test (*χ* ^2^[degrees of freedom]) or Fisher's exact test when Cochran's restriction applied, and ordinal data was compared using the Mann‐Whitney Rank Sum test (z). Regression analyses were used to evaluate the associations between pathologies when taking sex and age at death into account. Odds ratios (OR) with 95%CI are reported. Spearman's correlation (ρ) coefficient was used to calculate the correlation strength between the Braak stage and tangle stage (reported above). α was set at 0.05. Data were analyzed using STATA14 software (Stata Corporation 2015, Texas, USA).

## Results

Table 1 presents the main sociodemographic factors of the whole cohort (n = 672), and separately for CFAS (n = 437) and CC75C (n = 235). Out of the 672 cases 64% (n = 427) were female. The mean age at death was 88.6 ± 6.8 years. By using literature‐based criteria for HS‐Aging in CC75C, eleven out of 235 (5%) cases were identified.

**Table 1:** Sociodemographic characteristics.

	CFAS n = 437	CC75C n = 235	combined n = 672
Female, n (%)	262 (60%)	165 (70%)	427 (63%)
Age at death, mean ± standard deviation	87.0 ± 7.2	91.4 ± 4.7	88.6 ± 6.8

CFAS = Cognitive Function and Ageing Study, CC75C = Cambridge City over‐75s Cohort, n = number.

### Hippocampal neuron loss prevalence

In this population‐based cohort, 116 out of 672 (17%) cases displayed any hippocampal CA neuron loss as captured by our protocol. Table 2 summarizes the distributions of different neuron loss severities and locations by hippocampal subfield. Cases with neuron loss in several subfields are recorded for each category. Most frequently, neuron loss was observed in CA1 (n = 108, 16% of all cases). Only 16 (2%) of all cases had neuron loss recorded in other CA areas than CA1. Under half of these presented with focal neuron loss in only one sector – five cases with focal neuron loss in CA2, one in CA3, and one in CA4. Nine cases had neuron loss in multiple CA subfields.

**Table 2:** Numbers of cases with hippocampal neuron loss.

	<1 N/fov	1–5 N/fov	6–10 N/fov	11–15 N/fov	n
**CA4**					
Focal	0	0	2 (*1)	0	2 (*1)
Sub‐complete	2	0	0	0	2
Complete	1	1	3	0	5
**CA3**					
Focal	0	0	0	2 (*1)	2 (*1)
Sub‐complete	1	0	0	1	2
Complete	2	1	1	0	4
**CA2**					
Focal	1 (*1)	1	4 (*4)	0	6 (*5)
Sub‐complete	0	0	0	1	1
Complete	0	0	0	0	0
**CA1**					
Focal	16 (*15)	9 (*9)	21 (*21)	11 (*11)	57 (*56)
Sub‐complete	6 (*6)	6 (*6)	4 (*3)	6 (*5)	22 (*20)
Complete	10 (*7)	8 (*6)	7 (*7)	4 (*4)	29 (*24)

N = neuron, fov = field‐of‐view, n = number, (*) neuron loss only in the respective subfield.

### TDP‐43 pathology prevalence

pTDP‐43 immunostaining was available for hippocampal sections of 642 of the total 672 cases (96%). Across CC75C and CFAS, 33% of cases (n = 209/642) had pTDP‐43 inclusions in the hippocampus or entorhinal cortex (hippocampus: n = 193/639, 30%; entorhinal cortex: n = 167/627, 27%). 30% of the cohort (n = 192/642) had pTDP‐43 neurite pathology in the hippocampus or entorhinal cortex (hippocampus: n = 178/639, 28%; entorhinal cortex: n = 152/627, 24%).

Participants with TDP‐43 pathology in the hippocampus or entorhinal cortex were significantly older than those without [90.6 (±6.1) vs. 87.4 (±7.0) years]; [*t*(626) = −5.87, 95%CI: −4.20; −2.08, *P* < 0.001].

### HS‐aging diagnosis

All of the eleven HS‐Aging cases identified in CC75C by initial screening, were captured within the neuron loss category “no more than five neurons per fov in over half of the CA1 fov” at 200x magnification. No further cases of the reminder CC75C cohort, which were not initially evaluated as HS‐Aging, reached this level of neuron loss with neuron loss severity being ≤5 neurons/fov in an extent of ≥50% of CA1 fov. When these criteria were applied to the CFAS cohort, 23/437 (5%) HS‐Aging cases were identified. In all cases the neuron loss involved either the whole CA1 or its middle and distal thirds with relative sparing of CA1 neurons close to CA2. For confirmation, all cases which were not associated with vascular lesions and showed either neuron loss specific to CA1 or neuron loss extent wide enough to fulfil the proposed criteria for HS‐Aging were re‐evaluated, but no additional case fulfilled the criteria.

Five CC75C/CFAS cases that had neuron loss in multiple CA areas, also met the HS‐Aging CA1 neuron loss threshold. Four of these five cases were associated with vascular lesions, and one corresponded to epilepsy‐associated hippocampal sclerosis with dentate granular cell dispersion, hypertrophic neurons in CA4, and patchy neuron loss in CA4, CA3 and CA1. These cases were excluded from HS‐Aging diagnosis.

The HS‐Aging criteria also required that ischemic events in CA1 did not explain the severe neurons loss. One such case presented with a cavitary lesion involving the whole CA1, and the remains of a minor hemorrhage were visible at the subicular end of CA1 was thus not considered as a HS‐Aging case.

### HS‐aging sociodemographic characteristics

HS‐Aging cases had a significantly higher age at death (92.6 ± 5.6 years) compared to the remainder cohort (88.4 ± 6.8 years; *t*[670] = −3.56, 95%CI: −6.52; −1.89, *P* < 0.001), and a significant higher frequency of females (88% vs. 62%, *χ* ^2^(1) = 9.43, *P* = 0.002). Both age at death and sex remained significantly predictive of HS‐Aging when considered in logistic regression (sex: OR:3.54, 95%CI: 1.21–10.33; age: OR:1.10, 95%CI: 1.03–1.17).

### TDP‐43 pathology and HS‐aging

TDP‐43 staining was available for 33 out of 34 HS‐Aging cases. In one case, the entorhinal cortex was evaluated but not the hippocampus, because the section was cut too anterior.

**Figure 3:**
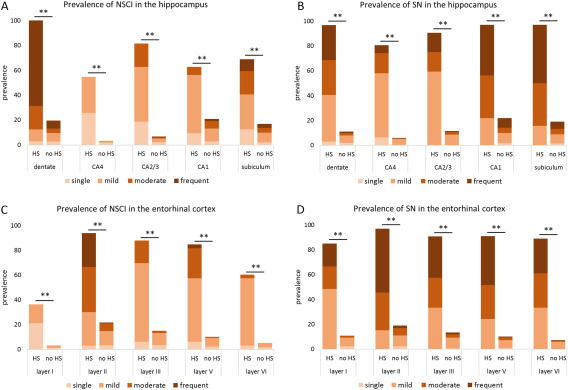
TDP‐43 inclusions and neurites pathology prevalence and severity in the hippocampus and entorhinal cortex by HS‐Aging status. All HS‐Aging cases were positive for dentate inclusions (A) as well as neurites (B) were significantly more prevalent and severe in all hippocampal regions compared to non‐HS‐Aging cases. Analogous, entorhinal cortex inclusions (C) and neurites (D) prevalence and severity were significantly higher in HS‐Aging than the remainder cohort. **P < 0.001, NSCI = TDP‐43‐positive neuronal solid cytoplasmic inclusions; SN = TDP‐43‐positive short neurites; HS = hippocampal sclerosis of aging.

All HS‐Aging cases were positive for hippocampal pTDP‐43 pathology. Dentate inclusions were present in all HS‐Aging cases where assessments were possible. Figure 3A presents the prevalence and severity of hippocampal inclusions for HS‐Aging and no‐HS‐Aging cases. Even though neurons are rare in HS‐Aging CA1, CA1 inclusions were present in two thirds of the cases. Compared to the remainder cohort, HS‐Aging was highly significantly associated with inclusions in all hippocampal areas (dentate: 100% vs. 20%, *χ* ^2^(1) = 109.36, *P* < 0.001; CA4: 55% vs. 3%, Fisher's exact, *P* < 0.001; CA2/3: 81% vs. 7%, Fisher's exact, *P* < 0.001; CA1: 63% vs. 21%, *χ* ^2^(1) = 29.92, *P* < 0.001; subiculum: 69% vs. 17%, *χ* ^2^(1) = 51.59, *P* < 0.001). In all hippocampal areas, also the severity of inclusions was significantly higher in HS‐Aging cases than in the reminder cohort (dentate: *z* = −11.41, *P* < 0.001; CA4: *z* = −12.04, *P* < 0.001; CA2/3: *z* = −13.45, *P* < 0.001; CA1: *z* = −4.99, *P* < 0.001; subiculum: *z* = −7.01, *P* < 0.001) (Figure 3A).

Hippocampal neurites were present in 32/33 HS‐Aging cases. All the positive HS‐Aging cases showed neurites in the dentate, CA1 and subiculum (Figure 3B). Analogous to inclusions, neurite frequencies in HS‐Aging cases were highly significantly different from the reminder cohort in all hippocampal areas (dentate: 97% vs. 11%, Fisher's exact, *P* < 0.001; CA4: 81% vs. 6%, Fisher's exact, *P* < 0.001; CA2/3: 91% vs. 11%, *χ* ^2^(1) = 145.03, *P* < 0.001; CA1: 97% vs. 22%, *χ* ^2^(1) = 89.93, *P* < 0.001; subiculum: 97% vs. 19%, *χ* ^2^(1) = 103.27, *P* < 0.001). In all hippocampal locations, the TDP‐43 neurites of HS‐Aging cases were significantly more severe than compared to the reminder cohort (dentate: *z* = −13.62, *P* < 0.001; CA4: *z* = −13.72, *P* < 0.001; CA2/3: *z* = −12.18, *P* < 0.001; CA1: *z* = −9.88, *P* < 0.001; subiculum: *z* = −10.82, *P* < 0.001) (Figure 3B).

Results for the entorhinal cortex inclusions and neurites were analogous to the hippocampus. Figure 3C illustrate the prevalence and severity of inclusions by layer for HS‐Aging compared to non‐HS‐Aging cases, and Figure 3D shows TDP‐43 neurites. Entorhinal cortex inclusions significantly associated with HS‐Aging [97% vs. 23%, *χ* ^2^(1) = 88.19, *P* < 0.001], as well as entorhinal cortex neurites [97% vs. 20%, *χ* ^2^(1) = 100.32, *P* < 0.001].

When controlling for sex and age at death in logistic regression, the association between TDP‐43 pathologies and HS‐Aging remained highly significant (hippocampal neurites: OR:85.68, 95%CI: 11.53–636.61; entorhinal cortex inclusions: OR:93.13, 95%CI: 12.53–692.34; entorhinal cortex neurites: OR: 109.86, 95%CI: 14.81–815.05).

### TDP‐43 and extensive CA1 neuron loss not fulfilling the criteria for HS‐aging

The case with ischemic complete severe CA1 neuron loss did not show hippocampal or entorhinal cortex TDP‐43 inclusions or neurites. In contrast, all evaluable cases with less severe neuron loss than HS‐Aging, but in over half of CA1 fields of view, no neuron loss in other CA areas, and no vascular lesions (n = 8), presented with both TDP‐43 inclusions and neurites in the hippocampus and entorhinal cortex.

### Focal CA1 neuron loss distribution

To identify possible further patterns of hippocampal neuron loss, focal CA1 neuron loss without neuron loss in other CA areas was evaluated in more detail. Table 3 presents the distribution of CA1 neuron loss only, by severity and location within CA1. Out of 56 cases, neuron loss was located in the proximal third of CA1 bordering CA2 in 11 cases, in the CA middle part in 13 cases, and in the distal end of CA1 in 32 cases. In 20 (36%) cases, CA1 focal neuron loss was associated with vascular pathology.

**Table 3:** Focal CA1 neuron loss characteristics, excluding cases with neuron loss in other CA areas.

CA1 location	<1 N/fov		1–5 N/fov		6–10 N/fov		11–15 N/fov		n
	n = 15		n = 9		n = 21		n = 11		
	nv	v	nv	v	nv	v	nv	v	
Beginning	1	3	1	2	1	2	1	0	11
Middle	1	3	3	1	1	2	1	1	13
End	4	3	1	1	13	2	8	0	32

N = neuron; fov = field of view; n = number; v = vascular; nv = non‐vascular.

**Figure 4:**
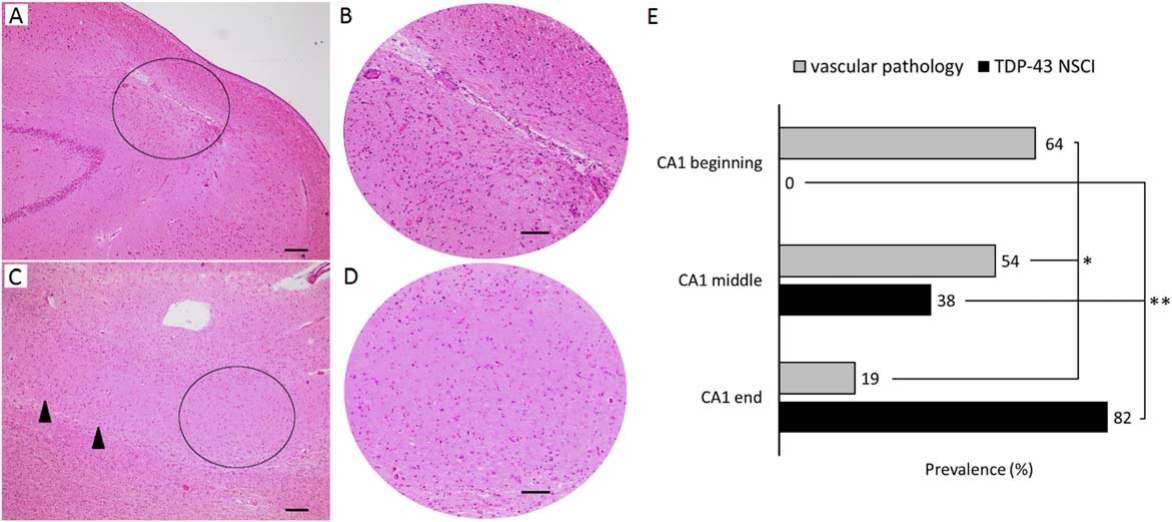
*Focal neuron loss characteristics in different locations of the CA1 sector*. (A) Focal neuron loss due to a microinfarct in the beginning of the CA1. The circle marks (B), one field‐of‐view at 200x magnification showing a localized, well circumscribed complete loss of neurons, and a glial scar. (C) Focal neuron loss in the subicular end of CA1, possibly associated with neurodegeneration. Neurons are preserved more proximal of the lesion (arrowheads). The circle marks (D), one field‐of‐view at 200x magnification showing disseminated gliosis and severe neuron loss, without indications of a microinfarct. Scale bars: (A, C): 200 μm; (B, D): 100 μm. (E) Among cases with neuron loss, lesions in the CA2‐bordering third of CA1 are most likely affected with ischemia (*P = 0.009), whereas neuron loss with TDP‐43 inclusions associates with the subicular end of CA1 (**P < 0.001). NSCI = neuronal solid cytoplasmic inclusions.

The beginning, middle and end sector of CA1 with focal neuron loss were differentially affected by vascular pathology [n = 7/11 (64%), n = 7/13 (54%) and n = 6/32 (19%) respectively; Fisher's exact, *P* = 0.008, Figure 4E]. When comparing focal neuron loss between CA1 subfields (two by two comparisons), we demonstrated that focal neuron loss near CA2 was significantly more frequently related with well demarcated scar‐like gliosis (Figure 4A,B) than the subicular end of CA1 (64% vs. 19%, Fisher's exact, *P* = 0.009). Results were similar when comparing lesions in the middle third to those in the subicular end of CA1 (54% vs. 19%, Fisher's exact, *P* = 0.025). Vascular lesions were equally common in focal neuron loss bordering CA2 as in the middle CA1 section (64% vs. 54%, Fisher's exact, *P* = 0.473). In contrast, focal neuron loss in the subicular end of CA1 showed disseminated diffuse neuron loss pattern with less aggressive gliosis than focal lesions bordering CA2 (Figure 4C,D).

### Focal CA1 neuron loss and TDP‐43

TDP‐43 immunostaining was possible in 51 of 56 cases with focal CA1 neuron loss. Non‐vascular CA1 focal neuron loss was significantly associated with inclusions: 32% (n = 6/19) of the cases with a vascular lesion presented with TDP‐43 inclusions, whereas 69% (n = 22/32) of the cases with focal neuron loss without recorded ischemic pathology were positive for TDP‐43 inclusions‐ and/or neurites (*χ* ^2^[1] = 6.65, *P* = 0.01).

TDP‐43 pathology was also strongly associated with the location of CA1 focal neuron loss: none of the ten cases that had a focal CA1 lesion bordering CA2 presented with inclusions or neurites, whereas 39% (n = 5/13) of the cases with a focal lesion in the middle third of CA1 were positive for inclusions (Fisher's exact, *P* = 0.038) and 31% (n = 4/13) for neurites (Fisher's exact, *P* = 0.081). 82% (n = 23/28) of the cases with a focal lesion in the CA1 sector bordering the subiculum showed TDP‐43 inclusions (Fisher's exact, *P* < 0.001), and 79% (n = 22/28) neurites (Fisher's exact, *P* < 0.001) (Figure 4E).

All 22 cases with focal non‐ischemic CA1 neuron loss that were positive for hippocampal TDP‐43 inclusions also had inclusions in the entorhinal cortex.

### Neuron loss in CA4‐2 and TDP‐43

TDP‐43 staining was available in 14 cases out of the 16 cases which had any category of neuron loss in CA4, CA3 or CA2. Four of these (29%) were positive for hippocampal inclusions, not differing from the remainder population (30%, n = 189/625, Fisher's exact, *P* = 0.578). Equivalently, 4/14 (29%) cases with neuron loss in CA4‐2 showed hippocampal neurites, whereas cases without neuron loss in these areas had a comparable neurite prevalence of 28% (n = 174/625, Fisher's exact, *P* = 0.578). No difference was observed in the prevalence of entorhinal cortex inclusions or neurites between cases with CA4‐2 neuron loss and the remainder cohort (inclusions: 28%, n = 4/14 vs. 27%, n = 163/613, Fisher's exact, *P* = 0.537; neurites: 36%, n = 5/14 vs. 24%, n = 147/613, Fisher's exact, *P* = 0.235).

### HS‐aging and other pathologies

Vascular pathologies were prevalent in this cohort. HS‐Aging was not associated with any of the evaluated CERAD vascular pathologies compared to the remainder [atherosclerosis: 33% vs. 32%, *χ* ^2^(1) = 0.01, *P* = 0.917; arteriolar sclerosis: 81% vs. 75%, *χ* ^2^(1) = 0.53, *P* = 0.468; cerebral infarcts >10 mm: 61% vs. 52%, *χ* ^2^(1) = 0.52, *P* = 0.470; cerebral infarcts < 10 mm: 44% vs. 35%, *χ* ^2^(1) = 0.50, *P* = 0.478; temporal microinfarcts: 0% vs. 9%, Fisher's exact, *P* = 0.387; frontal microinfarcts: 20% vs. 8%, Fisher's exact, *P* = 0.195; parietal microinfarcts: 20% vs. 14%, Fisher's exact, *P* = 0.426; occipital microinfarcts: 20% vs. 15%, Fisher's exact, *P* = 0.474].

HS‐Aging was not significantly associated with CERAD score: 24% (n = 8/34) had a CERAD score of zero, 18% (n = 6/34) a score A, 41% (n = 14/24) a score B and 18% (n = 6/34) a score C (Fisher's exact, *P* = 0.142).

Eleven HS‐Aging cases (32%) presented with Braak stage 0‐II, ten cases (29%) with stages Braak III‐IV, and 13 cases (38%) with Braak V‐VI. The distribution differed significantly from the remainder population [*χ* ^2^(2)= 6.40, *P* = 0.041], with HS‐Aging showing a lower prevalence of Braak III‐IV stages and higher prevalence of Braak 0‐II and V‐VI stages compared to non‐HS‐Aging cases (Braak 0‐II: 28%, III‐IV: 49%, V‐VI: 22%). However, when controlling for sex and age at death in logistic regression analysis, Braak stage did not significantly predict HS‐Aging (OR: 1.08, 95%CI: 0.65–1.79).

### Focal CA1 neuron loss and other pathologies

Focal CA1 neuron loss did not associate significantly with any of the CERAD vascular pathologies, neither when comparing ischemia‐related focal CA1 neuron loss to non‐vascular neuron loss, nor when comparing neuron loss by location within CA1.

Focal neuron loss at the subicular end of CA1 was significantly associated with a higher Braak stage compared to neuron loss in the beginning of CA1 (Fisher's exact, *P* = 0.001). No case with neuron loss at the subicular end of CA1, had a Braak stage of 0‐II. However, hippocampal tangle severity did not show a significant association with the focal neuron loss location (Fisher's exact, *P* = 0.304). In contrast to the Braak stage, focal CA1 neuron loss proximal to CA2 compared to neuron loss at the subicular end of CA1 did not differ significantly in CERAD score (Fisher's exact, *P* = 0.171).

## Discussion

This study evaluates in detail the hippocampal neuron loss pattern and TDP‐43 pathology in a population‐based cohort of older people. We report end‐stage HS‐Aging to be characterized by quantifiable measures of neuron loss location, extent and severity in association with presence of dentate TDP‐43 inclusions (Table 4). We show that there are neuron loss patterns within the hippocampal CA1 in the older population associated with distinct pathologies: focal CA1 neuron loss bordering CA2 was predominantly associated with vascular lesions, whereas neuron loss in the subicular end of CA1 associated with TDP‐43 and a high Braak stage.

**Table 4:** Key pathological characteristics of end‐stage hippocampal sclerosis of aging.

Neuron loss	location:	CA1; ±subiculum; sparing of CA4–2
	Extent:	≥50% of CA1 fov at 200x magnification
	Severity:	≤ five neurons per fov
TDP‐43	Solid neuronal inclusions:	present in the dentate of all cases highly prevalent in the entorhinal cortex
	Dystrophic short neurites:	highly prevalent in the hippocampus and entorhinal cortex
Tau	No association with the Braak stage or CERAD plaque score	
Vascular	No association with atherosclerosis, arteriolar sclerosis, cerebral infarcts, hippocampal microinfarcts or cortical microinfarcts	

Fov = field of view; CERAD = Consortium to Establish a Registry for Alzheimer's Disease.

There are methodological limitations to this study that need to be considered. In a research setting, neuron loss within a given tissue or structure is typically assessed with stereological methods 49, 52, which provide accurate and consistent measures. However, because of the required equipment and time, stereology is not compatible with routine neuropathological diagnostic methods, or for application to cohorts of many hundreds of cases. Furthermore, stereology requires thick sections, thus limiting tissue availability for other purposes. Because of these considerations, we designed a protocol for capturing different hippocampal neuron loss patterns relying on routine hematoxylin‐eosin‐sections.

Although measures of vascular and tau pathology followed the CERAD protocol 33, different pathologists were responsible for brain examinations, leading to potentially subjective interpretations, and varying grades of precision and completeness of the form. Furthermore, staining methods may have changed since the start of the studies, enhancing the risk of variations in scorings. These measurement errors are present in all long term multi‐center pathological studies without inter‐rater evaluation. By following an internationally standardized protocol comparability between studies is maximized when keeping in mind its limitations. We complemented the lack of consistency in Braak staging by a measure based on the available hippocampal and cortical tangle scores which correlated significantly with the “standard” Braak stage, but recognize that this is not an ideal solution.

We may have underdiagnosed focal hippocampal lesions and HS‐Aging, as only one or a few sections from each hippocampus from one hemisphere were assessed. HS‐Aging is reported to also occur segmentally 24, and in a quarter of cases unilaterally 35, 53, 58. However, the size and nature of this large, truly population‐based cohort derived from population representative studies should mitigate this limitation, and the detected associations related to various neuron loss patterns in the hippocampus and presence of TDP‐43 pathology are considered reliable.

This study focused on presence/absence of Ammon's horn pyramidal neurons. Minor discrepancies in neuron loss scoring occurred, and reasons were discussed between SRKH, SH and TMP. These included variation in decisions on CA area borders between each other and especially between CA1 and the subiculum, and some difficulties in decisions on counting neuron‐like shapes as neurons or not. However, results of our inter‐rater evaluations indicated that the neuron counts in this study are reliable and reproducible.

This study provides important strengths to investigate its aims. CA1 neuron loss is the key characteristic of old‐age HS‐Aging, as initially described by Dickson *et al* 18, but little systematic research on the pattern and severity of neuron loss within HS‐Aging has been conducted before this work. Recently, presence of TDP‐43‐pathology in the hippocampus has been used as an additional diagnostic criterion 5, 34. Unfortunately, existing definitions of HS‐Aging are still diverse, and the potential variable aetiologias behind hippocampal neuron loss add to this confusion.

Our initial assessment of HS‐Aging in the CC75C cohort was done independently by two raters according to CA1 neuron loss and gliosis descriptions in literature 16, 18, 36, 43, and diagnosis was confirmed by an experienced neuropathologist. Because all three raters agreed on all the cases, the likelihood of false HS‐Aging case inclusion or exclusion is low. When these HS‐Aging cases were compared to hippocampal neuron loss scorings, a CA1 neuron loss threshold of “maximal five pyramidal neurons in each field‐of‐view in over half of the CA1 fields‐of‐view at x200 magnification” defined the proposed HS‐Aging in our study. Interestingly all these cases presented with hippocampal TDP‐43 pathology. Thus, in these two population‐based cohorts, key HS‐Aging characteristics comprise neuron loss selectivity (sparing of CA2‐4), ischemia (not present), severity (<5 neurons/field‐of‐view), extent (>50% CA1 fields‐of‐view) and TDP‐43 inclusions (present).

Selectivity of neuronal loss differentiates HS‐Aging from HS‐epilepsy: in HS‐Aging, hippocampal neuronal loss is seen in the CA1 and subiculum only, whereas in HS‐epilepsy neuronal loss often affects the CA3 and CA4, and the granular cell layer shows irregularities, while the subiculum is often not affected 6, 9, 10, 47. Although some studies on HS‐Aging explicitly state a sparing of CA2/3 in HS‐Aging diagnosis 16, 17, 24, 27, 31, 55, others include cases which present with neuron loss also in other hippocampal sectors than the CA1 and subiculum 15, 29, 43, 44, 57, 58. Frequently, the term “selective” is used for CA1 neuron loss and gliosis, but how potential lesions in other CA areas are considered is not further specified (for example 2, 7, 26, 35). A consensus study categorized hippocampal lesions into six types; type four, characterized by “complete neuronal loss from CA1 because of neurodegeneration” and type three, described as “patchy diffuse neuronal degeneration in CA sectors associated with or without neuronal lesions of neurodegenerative origin” were both suggested to be recognized as HS‐Aging 45. Cases with neuron loss outside the CA1 and subiculum may not necessarily reflect the same disease processes that result in total neuron loss specifically in CA1 in older people, supported by our results of them not significantly associating with TDP‐43 pathology. Noticeable neuron loss in CA4‐2 could be regarded as evidence against HS‐Aging.

The original description of HS‐Aging by Dickson *et al* include “one case [where] the lesion was more extensive and associated with partial cyst formation” 18. Cavitary lesions are discussed controversially in literature ‐ cases with CA1 cavitary lesions are either specifically included 36 or excluded 14, 42, 56, 58, or not addressed in HS‐Aging diagnosis. In this study, only one case in the CFAS cohort presented with an extensive cavitary lesion in CA1 and met the HS‐Aging neuron loss threshold, but was not regarded as HS‐Aging, because of the co‐occurrence of vascular features including the cavern, characteristic patterns of gliosis and vacuolization, sharp demarcation of the gliotic neuron loss area toward the neighboring tissue and presence of micro‐hemorrhages and hemosiderin‐containing macrophages. This case also lacked hippocampal TDP‐43 pathology as did all of the cases with focal vascular lesions bordering CA2. We also could not confirm an association between any assessed vascular pathologies – whether in hippocampus, temporal lobe or elsewhere – and HS‐Aging. Although it is theoretically possible that exclusion of vascular pathology from HS‐Aging definition may have biased this finding, only the one vascular case described above would have met the neuron loss criteria of HS‐Aging, and would therefore not have affected statistical analyses. Findings of this population‐based study suggest that classical cerebrovascular pathologies are not primarily linked to HS‐Aging. Furthermore, even though CA1 is indisputably predisposed to ischemic lesions by its vascularization anatomy, results from rodent and non‐human primate studies have shown that transient cerebral hypoperfusion leads primarily to neuron loss in the junction of CA1/CA2, whereas neurons in the subicular end of CA1 are not affected 40, 48. Considering these findings, our results suggest that classical vascular brain lesions or transient hypoperfusion are unlikely directly linked with development of HS‐Aging.

A characteristic frequently included in HS‐Aging definition in literature is the absence of other neurodegenerative pathologies possibly accounting for the CA1 neuron loss, or neuron loss being out of proportion to neurofibrillary tangle pathology 1, 3, 22, due to its association with hippocampal atrophy and CA1 neuron loss 46, 51. However, this kind of definition is likely to increase the subjectivity of HS‐Aging diagnosis and thus dependence on the pathologist's personal opinion. In our study, HS‐Aging cases did not show high hippocampal tangle scores, probably due to the very low number of neurons in the CA1. The stains used did not allow us to evaluate reliably the possible presence of the “ghost” tangles. Regardless of the hippocampal tau pathology, we do not assume HS‐Aging and Alzheimer's disease to be mutually exclusive. We intended to avoid selection bias based on subjective expectations of pathological associations, but rather to describe features capturing HS‐Aging irrespective of a potential additional Alzheimer's disease. This approach gives a possibility to test the relationship between other pathologies and the strictly defined HS‐Aging. This view is supported by the lack of association between the severe selective neuron loss seen in the context of HS‐Aging and parameters traditionally related with Alzheimer's disease. In contrast, in this unselected population‐based study, no case without TDP‐43 pathology – apart from one case with a hippocampal infarct – reached the neuron loss severity and distribution identified for HS‐Aging.

Because we used criteria for definitive severe (end‐stage) HS‐Aging in CC75C according to most common descriptions in literature 16, 18, 36, 43, it is likely, that potential “pre‐HS‐Aging” cases with less neuronal loss were not categorized as HS‐Aging. Our finding of TDP‐43 pathology in all cases that were captured with our neuron loss evaluation protocol for extensive CA1 neuron loss, but who did not fulfill the criteria for HS‐Aging, is suggestive of this situation. However, the strict HS‐Aging criteria minimized false‐positive cases in the HS‐Aging category, and in association with the more detailed neuron loss pattern evaluation and TDP‐43 assessment allowed us to generate hypotheses on their association.

Interestingly, focal neuron loss characteristics differed significantly depending on their location within CA1: lesions bordering CA2 presented more frequently with a well‐demarcated area of severe neuron loss and/or glial scar, that is, findings of likely vascular lesions. None of the cases with CA1 lesions bordering CA2 presented with TDP‐43 or neurites. Our findings are in line with the location of hypoxic lesions reported in animal studies 40, 48, as well as results reported by Hatanpaa *et al* 23, who observed epilepsy‐associated and ischemic injury to be common in the proximal and middle CA1.

In contrast, focal neuron loss in CA1 bordering the subiculum was mostly diffuse with ill‐defined borders, and was less severe. Based on the morphology and its strong association with hippocampal TDP‐43 pathology, this focal neuron loss in the end of CA1 is hypothesized to be of primary neurodegenerative origin. In line with these results, Hatanpaa *et al* 23 described severe neuron loss in FTLD to be consistently located in the distal CA1. Our findings on more extensive CA1 neuron loss reflect the pattern observed in focal lesions.

Our pathological data are cross‐sectional and no direct conclusions on neuron loss progression can be made. However, in the older population there seems to be a specific pattern of neurodegeneration associated with TDP‐43 most commonly affecting the subicular end of CA1, while neurons close to CA2 are relatively spared. It can be hypothesized, that neuron populations within different CA1 subfields differ in their vulnerability to vascular and primary neurodegenerative damage. Based on these findings we hypothesize that HS‐Aging progresses from focal diffuse neuron loss in the subicular end of CA1 toward CA2. Longitudinal studies will be required to investigate this hypothesis further and biochemical work is necessary to identify possible features and differences in respective etiological pathways and to explore the roles of vascular disease, excitotoxicity, cytoskeletal changes and TDP‐43 in neuronal loss and HS‐Aging.

In summary, we describe neuron loss and TDP‐43 pathology characteristics in HS‐Aging, which could be used as objective end‐stage HS‐Aging criteria, enhancing consistency and comparability between future studies on this disorder. We present distinct patterns of primary neurodegenerative and vascular hippocampal neuron loss, which may contribute to understanding the specific vulnerability of different hippocampal neuron populations to neurodegenerative changes and aging. We suggest a neurodegenerative neuron loss progression from “pre‐HS‐Aging,” starting at the subicular end of CA1 in association with TDP‐43 pathology, to HS‐Aging. Further research is needed to explore these hypotheses.
